# Impact of genotype on endocrinal complications of Children with Alpha-thalassemia in China

**DOI:** 10.1038/s41598-017-03029-9

**Published:** 2017-06-07

**Authors:** Hong-Cheng Luo, Qi-Sheng Luo, Fu-Gao Huang, Chun-Fang Wang, Ye-Sheng Wei

**Affiliations:** 1 Department of Clinical Laboratory, the Affiliated Hospital of Youjiang Medical University for Nationalities, Baise, 533000 Guangxi China; 2 Department of neurosurgery, the Affiliated Hospital of Youjiang Medical University for Nationalities, Baise, 533000 Guangxi China; 3 Department of Ultrasound, the Affiliated Hospital of Youjiang Medical University for Nationalities, Baise, 533000 Guangxi China

## Abstract

Alpha-thalassemia occurs with high frenquency in China. Four common α-globin gene deletion mutations (–SEA, -α3.7, and -α4.2, Haemoglobin Constant Spring (CS) mutation) were identified in Chinese patients. Individuals with alpha-thalassemia syndrome are more often of children. However report on endocrinal complications in children with alpha thalassemia in China are still absent. The present study aimed to investigate the impact of genotype on endocrinal complications in Chinese children. Association analysis between genotype and endocrinal compliaction development was conducted on 200 patients with 200 healthy controls. Hypogonadism was found to be the most prominent endocrinal complications (84.0%) leading to the growth retardation, hypogonadism, diabetes mellitus, hypothyroidism and hypoparathyroidism whose incidence were significantly higher in pateints. (α^CS^α/–SEA) was the main genotype of Alpha thalassemia identified in the patients (37.5%), and patients with the (-α4.2/–SEA) genotype had a higher prevalence of hypogonadism, diabetes mellitus and hypoparathyroidism (P = 0.001, P = 0.001, P < 0.001, respectively).

## Introduction

Alpha-thalassemia (α-thalassemia) is caused by deletions or point mutations of the alpha-globin gene due to the complexity and diversity of genetic defects. The severity of the clinical phenotype of α-thal is diverse. Patients with severe α-thalassemia require frequent red blood cell transfusion for survival. As a result, many complications will occur in patients on regular blood transfusion with iron chelating therapy. Complications of α-thalassemia mainly result from chronic hemolysis and tissue hypoxia, causing iron overload and multiple organ dysfunction^1^.

A-thalassemia is a serious health problem worldwide, especially in Mediterranean areas, Southeast Asia and Southern China^2–4^. Guangxi Province is located in the southwest of China where the incidence of thalassemia is 24.51%^5^. However, in the past decades, data on diagnose and treatment of α-thalassemia or related complications in children are still absent. In this study typical physical exam findings growth retardation, hypogonadism, thalassemic bone deformities, diabetes mellitus^6–9^ were included to identify the association between four genotype (SEA, -α3.7/–SEA, -α4.2/–SEA, α^CS^α/–SEA) and endocrine complications in children with α-thalassemia.

## Materials and Methods

### General

Two hundred Children (126 males and 74 females) with mean age of 9.64 ± 1.15 years (range, 3–12 years). Who were registered in The Affiliated Hospital of Youjiang Medical College for Nationalities from the period January 2010 to June 2016 were included in this research. α- thalassemia children were characterized with one of the genetype of SEA, -α3.7/–SEA, -α4.2/–SEA or α^CS^α/–SEA. Which was identified by the DNA sequencing technique. The basic clinical information collected included Average Hematological Parameters of diagnosis, gender, age, age of start transfusion, age of start chelation, frequency of transfusion and related compliance.

The study was approved by the Ethical committee of Youjiang Medical College for Nationalities and written informed consent was obtained from the subjects. The study was in compliance with the Helsinki declaration.

We selected 200 cases of the same age group as a control group for research (113 males and 87 females, 9.35 ± 1.56 years, range, 3–12 years). The criteria for the control group are as follows: All individuals have a normal level of mean corpuscular volume (MCV) > 82.6 < 99.1fl, and mean corpuscular hemoglobin (MCH) > 26.9 < 33.3. A normal level of HbA (between 85% and 97.5%) and HbA2 (between 2.5% and 3.5%), the Normal levels serum ferritin, no one suffering from hemolytic anemia and malnutrition anemia. No cardiovascular and blood infectious d disease. Its family without hypertension, diabetes. All the control group also were diagnosed by the DNA sequencing technique, no one suffers from six common α- thalassemia (–SEA, -α3.7, -α4.2, α^CS^α, α^WS^α, α^QS^α) and seventeen β- thalassemia (17 M/N, CD41-42M/N, -28M/N, -29M/N, 31 M/N, -32M/N, 43 M/N, 654 M/N, -30M IVS-I-1M, IVS-I-5M 14–15 M, 27/28 M, 71–72 M/N, ΒeM/N, CAMP, IntM), which the common type of thalassemia in chinese people.

#### Physical examination including

Red blood cells (RBC), Hemoglobin (HGBg/l), Mean corpuscular volume (MCV/fl), Mean hemoglobin content (MCH/pg), Mean hemoglobin concentration (MCHC), basal growth hormone, estradiol (in females) and testosterone (in males), thyroid-stimulating hormone (TSH), FT3, FT4, serum calcium concentration, serum phosphate and parathyroid hormone. Alpha globin mutations were analyzed using gap-PCR and reverse-hybridization assay according to the manufacturer.

#### Classification of patients according to genotype

Children were divided into four groups according to their genotype based on the α-globin gene production. Group1–4: (SEA) deletions, (−3.7 kb merge SEA) deletions, (−4.2 kb merge SEA) deletions and (CS mutations merge SEA) deletions.

#### Definitions

Short stature was defined as patient height **>**2 standard deviation below the mean for age, gender and ethnicity^10^. Short stature was evaluated by Children’s Health Rehabilitation Center (Affiliated Hospital of Youjiang Medical College for Nationalities, Guangxi, China).

Hypogonadism was defined as low testosterone (in males or oestradiol (in females) level or subjects who had received testosterone or oestradiol therapy.

Patients were diagnosed with diabetes mellitus based on WHO criteria or history of insulin therapy or oral antidiabetic therapy according to American Diabetes Association, World Health Organization Criteria and National Diabetes Health Group 1979.

Hypothyroidism was defined according to TSH/FT3, FT4 or based on the history of treatment with levothyroxine for previously diagnosed hypothyroidism. Hypoparathyroidism was defined as low serum calcium and low serum parathyroid hormone concentration, with increased serum phosphate.

A hemoglobin level of less than 90 (g/L) was the standard for initiating transfusion in children with severe thalassemia. Infection, growth retardation, diabetes mellitus, hypogonadism, hypothyroidism, hypoparathyroidism or other complications in thalassemia children, were the indications for transfusion at a relatively high level of haemoglobin.

### Statistical analysis

SPSS13.0 (SPSS, Inc., IL, USA) was used to conduct statistical analysis. ***χ*** ^**2**^ test or Fisher’s exact test was used for comparation between different groups. Measurement data were represented as mean ± standard deviation ($\bar{x}\pm s$), and categorical data were represented as ***χ*** ^**2**^. *P* < 0.05 and *P* < 0.001 were considered to indicate statistically significant differences.

## Results

### Patient characteristics

All the patients were recruited from Affiliated Hospital of Youjiang Medical College for Nationalities, Guangxi, China. The patients (126 males and 74 females) had a mean (SD) age of (9.64 ± 1.15) years. Hypogonadism was the most prominent endocrinal complications in patients (84.0%), followed by growth retardation (68.5%) and hypoparathyroidism (14.5%). A total 70.5% of patients start to use chelation in 3 years old. There was no significant difference in RBC, MCV, MCH and MCHC among the four groups (*P* > 0.05). Clinical Average Hematological Parameters were summarized in (Table 1).

**Table 1:** Clinical Average Hematological Parameters of the Study Population ($\bar{x}\pm s$).

Genetype	n	RBC (*10^12^/L)	HGB (g/L)	MCV (fl)	MCH (pg)	MCHC (g/L)	Serum ferritin (ng/ml)
SEA	65	5.21 ± 1.08	85.69 ± 23.44	62.43 ± 7.31	19.11 ± 2.29	305.11 ± 14.70	356.17 ± 25.76
-α^3.7^/–SEA	37	5.09 ± 0.82	84.43 ± 15.63	56.28 ± 6.56	16.70 ± 1.67	299.61 ± 21.01	976.58 ± 79.11
-α^4.2^/–SEA	23	5.32 ± 0.85	80.88 ± 14.67	52.49 ± 2.44	16.74 ± 2.90	294.50 ± 15.02	997.37 ± 78.69
α^CS^α/–SEA	75	4.11 ± 0.96	79.17 ± 18.89	68.63 ± 8.38^a,b^	18.64 ± 2.03	270.00 ± 25.01	1023.69 ± 81.55
Reference		3.50~5.50	110.00~160.00	80.00~100.00	27.00~34.00	320.00~360.00	15.00~250.00

RBC, red blood cell; HGB, haemoglobin; MCV, mean corpuscular volume; MCH, mean corpuscular haemoglobin; MCHC, mean corpuscular haemoglobin concentration; SEA: the Southeast Asian deletion; -α3.7, rightward deletion; -α4.2, leftward deletion; CS, Hb Constant Spring.

a:compared with -α4.2/–SEA, P < 0.001;

b:compared with -α3.7/–SEA, P < 0.001.

**Note**: RBC, HGB, MCV, MCH and MCHC were the average hematological parameters level before the first blood transfusion.

### Genetype of Thalassemia and endocrinal complications

Two major genetype identified in the Alpha thalassemia patients were α^CS^α/–SEA (37.5%, 38.1% of males and 36.5% of females) and SEA (32.5%), followed by -α3.7/–SEA (18.5%) and -α4.2/–SEA (11.5%). A total of 94.1% of patients with the α^CS^α/–SEA genotype started earlier transfusion (≤3 year), 77.3% of patients received frequent transfusion (every 4–5 weeks) and 68.0% started earlier iron chelators (>3 years). In addition, patients with the α^CS^α/–SEA genotype had a higher prevalence of growth retardation (92%), and patients with the -α4.2/–SEA genotype had a higher prevalence of hypogonadism and diabetes mellitus (100% and 73.9%, respectively) (Table 2).

**Table 2:** Association between patient genotype and endocrinal complications.

Characteristics	Patients	SEA (n = 65)	-α^3.7^/–SEA (n = 37)	-α^4.2^/–SEA (n = 23)	α^CS^α/–SEA (n = 75)	*P*-value
Gender						
Male	126	31.7%	19.0%	11.1%	38.1%	0.98
Female	74	33.8%	17.6%	12.2%	36.5%	
Age of start transfusion (years)						
≤3	117	22.2%	13.7%	10.3%	53.8%	<0.001
>3	83	47.0%	25.3%	13.3%	14.5%	
Frequency of transfusion (weeks)						
Every 2–3	98	45.9%	19.4%	17.3%	17.3%	<0.001
Every 4–5	102	19.6%	17.6%	5.9%	56.9%	
Age of start chelation (years)						
≤6	59	27.1%	15.3%	16.9%	40.7%	0.31
>6	141	34.8%	19.9%	9.2%	36.2%	
Growth retardation						
Negative	63	50.8%	25.4%	14.3%	9.5%	<0.001
Positive	137	24.1%	15.3%	10.2%	50.4%	
Hypogonadism						
Negative	32	62.5%	15.6%	0.0%	1.9%	0.001
Positive	168	26.8%	19.0%	13.7%	40.5%	
Diabetes mellitus						
Negative	186	33.9%	18.3%	9.1%	38.7%	0.001
Positive	14	14.3%	21.4%	42.9%	21.4%	
Hypothyroidism						
Negative	174	36.8%	14.4%	8.6%	40.2%	<0.001
Positive	26	3.8%	46.2%	30.8%	19.2%	
Hypoparathyroidism						
Negative	171	38.0%	16.4%	9.4%	36.3%	<0.001
Positive	29	0.0%	31.0%	24.1%	44.8%	

### Growth retardation in patients

Growth retardation was identified in 75.2% of patients (≥6 years old) and 61.1% of patients (<6 years old), and no significant difference was identified between males and females. A total of 40.9% of patients with growth retardation started earlier blood transfusion (≤3 year), 69.3% received frequent transfusion (every 4–5 weeks), 89.8% started iron chelation (>3 years) and 17.5% were poor compliant (Table 3).

**Table 3:** Association between growth retardation and each of the demographic, frequency of transfusion, age of start transfusion, Age of start chelation, compliance.

Characteristics	Patients, n	Growth retardation (n = 200)		*P*-value
		Negative (n = 63)	Positive (n = 137)	
Gender				
Male	126	31.0%	69.0%	0.83
Female	74	32.4%	67.6%	
Age (years)				
≥6	105	24.8%	75.2%	0.03
<6	95	38.9%	61.1%	
Frequency of transfusion (weeks)				
Every 2–3	98	57.1%	42.9%	<0.001
Every 4–5	102	6.9%	93.1%	
Age of start transfusion (years)				
≤3	117	52.1%	47.9%	<0.001
>3	83	2.4%	97.6%	
Age of start chelation (years)				
≤6	59	76.3%	23.7%	<0.001
>6	141	12.8%	87.2%	
Compliance, %				
<60	43	44.2%	55.8%	0.04
≥60	157	28.0%	72.0%	

### Hypogonadism in patients

Hypogonadism was identified in 83.8% of patients (≥6 years old) and 84.2% of patients (<6 years old), and there was significant difference between males and females (*P* < 0.001). A total of 67.3% of patients with hypogonadism started earlier transfusion (≤3 years), 51.8% of them received frequent transfusion (every 2–3 weeks). 76.2% of patients with hypogonadism started iron chelation (>3 years) and 12.5% had a poor compliance (Table 4).

**Table 4:** Association between hypogonadism and each of the demographic, frequency of transfusion, age of start transfusion, Age of start chelation, compliance.

Characteristics	Patients, n	Hypogonadism (n = 200)		*P*-value
		Negative (n = 32)	Positive (n = 168)	
Gender				
Male	126	23.0%	77.0%	<0.001
Female	74	4.1%	95.9%	
Age (years)				
≥6	105	16.2%	83.8%	0.94
<6	95	15.8%	84.2%	
Frequency of transfusion (weeks)				
Every 2–3	98	11.2%	88.8%	0.07
Every 4–5	102	20.6%	79.4%	
Age of start transfusion (years)				
≤3	117	3.4%	96.6%	<0.001
>3	83	33.7%	66.3%	
Age of start chelation (years)				
≤6	59	32.2%	67.8%	<0.001
>6	141	9.8%	90.8%	
Compliance, %				
<60	43	20.9%	79.1%	0.32
≥60	157	14.6%	85.4%	

### Diabetes mellitus in patients

Diabetes mellitus was identified in 14 patients and 71.4% of them were ≥6 years old with no significant difference identified between males and females. 92.9% of them received frequent transfusion (every 2–3 weeks), and 85.7% of patients with hypogonadism started iron chelation (>3 years) (Table 5).

**Table 5:** Association between diabetes mellitus and each of the demographic, frequency of transfusion, age of start transfusion, Age of start chelation, compliance.

Characteristics	Patients, n	Diabetes mellitus (n = 200)		*P*-value
		Negative (n = 186)	Positive (n = 14)	
Gender				
Male	126	95.2%	4.8%	0.11
Female	74	89.2%	10.8%	
Age (years)				
≥6	105	90.5%	9.5%	1.14
<6	95	95.8%	4.2%	
Frequency of transfusion (weeks)				
Every 2–3	98	99.0%	1.0%	0.001
Every 4–5	102	87.3%	12.7%	
Age of start transfusion (years)				
≤3	117	95.7%	4.3%	0.07
>3	83	89.2%	10.8%	
Age of start chelation (years)				
≤6	59	96.6%	3.4%	0.32
>6	141	91.5%	8.5%	
Compliance, %				
<60	43	88.4%	11.6	0.32
≥60	157	94.3%	5.7%	

### Hypothyroidism in patients

26 patients (15 males and 11 males) were diagnosed with hypothyroidism and no significant difference was identified between males and females. All of these patients started earlier transfusion (≤3 years). Most of the patients (88.5%) were more than 6 years older and 96.2% had a poor compliant (Table 6).

**Table 6:** Association between hypothyroidism and each of the demographic, frequency of transfusion, age of start transfusion, Age of start chelation, compliance.

Characteristics	Patients	Hypothyroidism (n = 200)		*P*-value
		Negative (n = 174)	Positive (n = 26)	
Gender				
Male	126	88.1%	11.9%	0.70
Female	74	85.1%	14.9%	
Age (years)				
≥6	105	78.1%	21.9%	<0.001
<6	95	96.8%	3.2%	
Frequency of transfusion (weeks)				
Every 2–3	98	79.6%	20.4%	0.004
Every 4–5	102	94.1%	5.9%	
Age of start transfusion (years)				
≤3	117	77.8%	22.2%	<0.001
>3	83	100.0%	0.0%	
Age of start chelation (years)				
≤6	59	67.8%	32.2%	<0.001
>6	141	95.0%	5.0%	
Compliance, %				
<60	43	41.9%	58.1%	<0.001
≥60	157	99.4%	0.6%	

### Hypoparathyroidism in patients

Hypoparathyroidism was identified in 29 patients and 82.8% of them were ≥6 years old, no significant difference was observed between males and females. All of these patients started earlier transfusion (≤3 years) and most of them had a poor compliant (Table 7).

**Table 7:** Association between hypoparathyroidism and each of the demographic, frequency of transfusion, age of start transfusion, Age of start chelation, compliance.

Characteristics	Patients	Hypoparathyroidism (n = 200)		*P*-value
		Negative (n = 171)	Positive (n = 29)	
Gender				
Male	126	87.3	12.7	0.35
Female	74	82.4	17.6	
Age (years)				
≥6	105	77.1	22.9	<0.001
<6	95	94.7	5.3	
Frequency of transfusion (weeks)				
Every 2–3	98	80.6	19.4	0.05
Every 4–5	102	90.2	9.8	
Age of start transfusion (years)				
≤3	117	75.2	24.8	<0.001
>3	83	100.0	0.0	
Age of start chelation (years)				
≤6	59	69.5	30.5	<0.001
>6	141	92.2	7.8	
Compliance, %				
<60	43	39.5	60.5	<0.001
≥60	157	98.1	1.9	

### Endocrine complication between case group and control group

There was no significant difference in the incidence of endocrine complication between male and female in case group and control group, alpha thalassemia patients are significantly more likely to have growth retardation, hypogonadism, diabetes mellitus, hypothy- roidism and hypoparathyroidism compared with controls (*P* < 0.001) (Table 8). The HGB level lower in patients (81.17 ± 15.23 g/L, range, 13~95 g/L) than control subjects (126.21 ± 17.65 g/L, range, 55~167 g/L). We also identified a significant difference between RBC and MCV indices in case group and control group (*P* < 0.001).

**Table 8:** Comparison of endocrine complications in patients with alpha thalassemia and control group.

Characteristics	Alpha thalassemia (n = 200)	Contral group (n = 200)	*P*-value
Gender			
Male	126 (63.0%)	113 (56.5%)	0.22
Female	74 (37.0%)	87 (43.5%)	
Growth retardation			
Negative	137 (68.5%)	195 (97.5%)	<0.001
Positive	63 (31.5%)	5 (2.5%)	
Hypogonadism			
Negative	168 (84.0%)	197 (98.5%)	<0.001
Positive	132 (16.0%)	3 (1.5%)	
Diabetes mellitus			
Negative	186 (93.0%)	198 (99.0%)	0.005
Positive	14 (7.0%)	2 (1.0%)	
Hypothyroidism			
Negative	174 (87.0%)	193 (96.5%)	0.001
Positive	26 (13.0%)	7 (3.5%)	
Hypoparathyroidism			
Negative	171 (85.5%)	199 (99.5%)	<0.001
Positive	29 (14.5%)	1 (0.5%)	

## Discussion

Thalassemia is a well-known inherited hematologic disorder caused by reduced or absence of globin production^11^. In China, this disease is prevalent in areas near the southern bank of the Yangtze River, such as Guangdong, Guangxi, Fujian and Yunnan Provinces^12–14^. Endocrine dysfunction is a frequent complication in thalassemic patients who are on regular blood transfusions. Iron overload has been considered to be the major cause of endocrine abnormalities of α-thalassemia^15^. Growth retardation, hypogonadism, diabetes mellitus and hypoparathyrodism represent the most common endocrinopathies in thalassemic patients^10^. In this study, we evaluates the impact of genotype on endocrinal complications of Children with Alpha- thalassemia in China and demonstrates that hypogonadism is the most frequent endocrine complication in α-thalassemia (84.0%), followed by growth retardation (68.5%) and hypoparathyroidism (14.5%).

Our survey showed that the MCV levels in group (α^CS^α/–SEA) were higher than those in group (-α3.7/–SEA) and group (-α4.2/–SEA)(*P* < 0.001, *P* < 0.001, respectively), there were no significant differences in RBC, HGB, MCH and MCHC levels among the four groups (*P* > 0.05), similar to the previous study by Zhu *et al*.^16^. Compared with the other three groups (α^CS^α/–SEA, -α3.7/–SEA, -α4.2/–SEA), the group SEA had a significant lower serum ferritin levels (*P* < 0.001, respectively), this may be due to patients with SEA genetype generally do not receive blood transfusion therapy frequently unless combined with iron deficiency anemia, vitaminD deficiency, infection caused by long-term malnutrition anemia. In consistent with report by Zhou Y. U. *et al*.^17^ no significant difference was observed among the three group (α^CS^α/–SEA, -α3.7/–SEA, -α4.2/–SEA) in Serum ferritin levels (*P* > 0.05, respectively).

In the present study, we found that the patients with the genetype of (α^CS^α/–SEA) had significant higher prevalence of growth retardation, hypogonadism (*P* < 0.001, *P* = 0.001, respectively). Just like previous report^18–20^ hypogonadism was identified as the most common endocrine complication in the patients (84.0%). Gender, age of start transfusion or start Chelation had a significant impact on hypogonadism development. However a lower prevalence of hypogonadism was found in some study^21, 22^, which were mainly attributed to difference in the economic status of patients, Physicians’ strategies to optimize chelation therapy, promoting compliance, educating patients and different ethnic^23–26^. The patients with the genetype of (-α4.2/–SEA) had a significantly higher prevalence of diabetes mellitus (*P* = 0.001). And there was no significant differences in the incidence of genotypes between males and females (*P* = 0.98).

Compared to the present study 68.5% of patients identified with growth retardation, Hattab, F. N. *et al*.^27^ found a higher prevalence of growth retardation (75.9%). This may be attributed to the difference in economy, most of the patients come in the latter study from poor families, received poor health care treatment, which resulted in multiple infections, thereby aggravating growth retardation or other potential endocrine complications development in Alpha- thalassemia during childhood. Futher more, the discrepancy of clinical manifestations may be impacted by genetic and environmental factors^28–30^. There was significant association between growth retardation and older year (≥6 years), earlier age of start transfusion, chelation, frequency of blood transfusion or poor compliance (*P* = 0.03, *P* < 0.001, *P* < 0.001, *P* < 0.001, *P* = 0.04, respectively). But there was no significant association between growth retardation and gender (*P* = 0.83).

In the present study, 7.0% of patients were diagnosed with diabetes mellitu, similar to 8.0% in report by Ong, C. K. *et al*.^31^. Several previous study have report a lower prevalence of diabetes mellitu, which ranged from 2.5% to 4.9%^32–34^, while Other had report a higer prevalence of diabetes mellitu, reaching 13% to 17.0%^35–37^. These discrepancies can be attributed to differences in the age of patients and severity of Hepatitis C virus infection, transfusion rates and chelation therapies, male sex, liver iron concentration^38, 39^. There was significant association between diabetes mellitu and frequency of blood transfusion (*P* = 0.001), but there was no significant association between diabetes mellitu and gender, age, age of start transfusion, chelation, frequency blood transfusion or compliance (*P* = 0.11, *P* = 1.14, *P* = 0.07, *P* = 0.32, *P* = 0.32, respectively).

Hypothyroidism was identified in 26 patients (13.0%), which was similar to the result reported by Eshragi, P. *et al*.^40^. While, other studies reported a lower prevalence of hypothyroidism, which ranged from 1.0% to 10.0%^41–43^. The results of different studies vary widely, these discrepancies can be attributed to differences in genotype of thalassemia, the age of patients or treatment protocols.

Hypogonadism (84.0%), growth retardation (68.5%) and hypoparathyroidism (14.5%) were the first and the most frequent endocrine complications diagnosed in our present study. Today, many patients can benefit from modern treatment, improve the quality of life of patients dut to adopting in early and regular chelation therapy. Therefore, prevention of the endocrine complications may be influenced by the improvement of medical diagnosis and treatment. Monitoring compliance is essential in such conditions.

There are a few limitations need to be mention here. Firstly, the sample size is small, and the age of these patients too early which may result in limited power. Secondly, the type of iron chelation used could not be figured out, rare genetype of α-thalassemia were not included in our study. Thirdly, none of the analyses take into account the age effect properly. The incomplete medical records could prevent us from identifying predictive complication. Further studies are needed on the complications of all α-thalassemic and older patients in the region.

In conclusion, our present study show that α^CS^α/–SEA, SEA, -α3.7/–SEA, and -α4.2/–SEA are the main genetype identified in α-thalassemia children in Guangxi Province, and hypogonadism, growth retardation and hypoparathyroidism are the most common endocrine complications in children with α-thalassemia.
